# Identification of patients with branch-duct intraductal papillary mucinous neoplasm and very low risk of cancer: multicentre study

**DOI:** 10.1093/bjs/znac103

**Published:** 2022-05-03

**Authors:** Domenico Tamburrino, Nicolò de Pretis, Enrique Pérez-Cuadrado-Robles, Laura Uribarri-Gonzalez, Zeeshan Ateeb, Giulio Belfiori, Patrick Maisonneuve, Gabriele Capurso, Giuseppe Vanella, Maria Chiara Petrone, Paolo Giorgio Arcidiacono, Yrjo Vaalavuo, Luca Frulloni, J. Enrique Dominguez-Muñoz, Pierre H. Deprez, Massimo Falconi, Marco del Chiaro, Stefano Crippa, Johanna Laukkarinen

**Affiliations:** Pancreatic Surgery Unit, Vita-Salute University, Pancreas Translational and Clinical Research Centre, San Raffaele Scientific Institute IRCCS, Milan, Italy; Department of Gastroenterology, Pancreas Institute, University of Verona, Verona, Italy; Department of Hepato-Gastroenterology, Cliniques Universitaires Saint-Luc, Brussels, Belgium; Department of Gastroenterology, Hôpital Européen Georges-Pompidou, Paris, France; Department of Gastroenterology, Complejo Hospitalario de Navarra, Pamplona, Spain; Department of Gastroenterology, Hospital Universitario de Santiago de Compostela, Santiago de Compostela, Spain; Pancreatic Surgery Unit, Division of Surgery, Department of Clinical Science, Intervention and Technology (CLINTEC), Karolinska Universitetsjukhuset i Huddinge, Huddinge, Sweden; Pancreatic Surgery Unit, Vita-Salute University, Pancreas Translational and Clinical Research Centre, San Raffaele Scientific Institute IRCCS, Milan, Italy; Division of Epidemiology and Biostatistics, IEO European Institute of Oncology IRCCS, Milan, Italy; Digestive and Liver Disease Unit, Sant’Andrea Hospital, Rome, Italy; Pancreato-Biliary Endoscopy and Endosonography Division, Pancreas Translational and Clinical Research Centre, San Raffaele Scientific Institute IRCCS, Vita-Salute San Raffaele University, Milan, Italy; Digestive and Liver Disease Unit, Sant’Andrea Hospital, Rome, Italy; Pancreato-Biliary Endoscopy and Endosonography Division, Pancreas Translational and Clinical Research Centre, San Raffaele Scientific Institute IRCCS, Vita-Salute San Raffaele University, Milan, Italy; Pancreato-Biliary Endoscopy and Endosonography Division, Pancreas Translational and Clinical Research Centre, San Raffaele Scientific Institute IRCCS, Vita-Salute San Raffaele University, Milan, Italy; Pancreato-Biliary Endoscopy and Endosonography Division, Pancreas Translational and Clinical Research Centre, San Raffaele Scientific Institute IRCCS, Vita-Salute San Raffaele University, Milan, Italy; Department of Gastroenterology and Alimentary Tract Surgery, Tampere University Hospital and Faculty of Medicine and Health Technology, Tampere University, Tampere, Finland; Department of Gastroenterology, Pancreas Institute, University of Verona, Verona, Italy; Department of Gastroenterology, Hospital Universitario de Santiago de Compostela, Santiago de Compostela, Spain; Department of Hepato-Gastroenterology, Cliniques Universitaires Saint-Luc, Brussels, Belgium; Pancreatic Surgery Unit, Vita-Salute University, Pancreas Translational and Clinical Research Centre, San Raffaele Scientific Institute IRCCS, Milan, Italy; Division of Surgical Oncology, Department of Surgery, University of Colorado, Anschutz Medical Campus, Denver, Colorado, USA; Pancreatic Surgery Unit, Vita-Salute University, Pancreas Translational and Clinical Research Centre, San Raffaele Scientific Institute IRCCS, Milan, Italy; Department of Gastroenterology and Alimentary Tract Surgery, Tampere University Hospital and Faculty of Medicine and Health Technology, Tampere University, Tampere, Finland

## Abstract

**Background:**

Different surveillance strategies for patients with low-risk branch-duct (BD) intraductal papillary neoplasm (IPMN) have been described. The aim of this study was to describe the natural history of low-risk BD-IPMN, and to identify risk factors for the development of worrisome features (WF)/high-risk stigmata (HRS) and of pancreatic malignancies.

**Methods:**

This was a multicentre retrospective study of patients with BD-IPMN who were under active surveillance between January 2006 and December 2015. Patients were eligible if they had a low-risk lesion and had a minimum follow-up of 24 months. Outcomes were development of WF/HRS or cytologically/histologically confirmed malignant IPMN.

**Results:**

Of 837 patients included, 168 (20 per cent) developed WF/HRS. At the end of the observation time, 132 patients (79 per cent) with WF/HRS were still under surveillance without progression to pancreatic cancer. Factors associated with the development of WF or HRS in multivariable analysis included localized nodules (*versus* diffuse: hazard ratio (HR) 0.43, 95 per cent c.i. 0.26 to 0.68), cyst size 15–19 mm (*versus* less than 15 mm: HR 1.88, 1.23 to 2.87) or at least 20 mm (*versus* less than 15 mm: HR 3.25, 2.30 to 4.60), main pancreatic duct size over 3 mm (*versus* 3 mm or less: HR 2.17, 1.41 to 3.34), and symptoms at diagnosis (*versus* no symptoms: HR 2.29, 1.52 to 3.45). Surveillance in an endoscopy-oriented centre was also associated with increased detection of WF or HRS (*versus* radiology-oriented: HR 2.46, 1.74 to 3.47).

**Conclusion:**

Conservative management of patients with low-risk BD-IPMN is safe and feasible.

## Teaser

Conservative management of patients with low-risk branch-duct intraductal papillary neoplasm is safe and feasible. A size of 15 mm at first examination seems to be associated with a low risk of progression.

## Introduction

In the general population, the prevalence of cystic neoplasms of the pancreas is around 8 per cent^1^. Most lesions are branch-duct (BD) intraductal papillary mucinous neoplasms (IPMNs), which are usually detected incidentally^2^. They have a more indolent behaviour than mixed-type or main-duct IPMNs. In 2006, international consensus guidelines^3^ incorporated non-operative management for low-risk asymptomatic IPMNs less than 30 mm in size, with negative cytology, and without nodules and main pancreatic duct (MPD) dilatation. Updates of the guidelines in 2012^4^ and 2017^5^ introduced two categories of risk for malignancy, namely worrisome features (WF) and high-risk stigmata (HRS), and surveillance was recommended for BD-IPMNs lacking these features. A similar approach was proposed in the 2018 European guidelines^6^, and has been validated by different studies^7,8^. International^5^ and European^6^ guidelines propose a different surveillance schedule for low-risk BD-IPMNs, but they are concordant in supporting lifetime observation unless patients become unfit for surgery. A more liberal approach has been suggested by the American Gastroenterological Association guidelines^9^, which include discontinuation of surveillance after 5 years in the absence of significant changes. Although lifetime surveillance is costly, a general recommendation for discontinuation after 5 years may be inappropriate^10,11^.Therefore, studying the progression of low-risk BD-IPMN is clinically relevant for a better definition of surveillance timing and possible discontinuation in selected patients. The aim of the present study was to describe the natural history of low-risk BD-IPMNs, to identify risk factors for the development of WF/HRS and pancreatic malignancies, and to eventually define a subgroup of patients at low or no risk of progression over time.

## Methods

### Study design and setting

This was a multicentre, retrospective study carried out under the auspices of Pancreas 2000, the official postgraduate educational and research programme of the European Pancreatic Club (http://www.pancreas2000.org). International centres that participated included: Division of Pancreatic Surgery, San Raffaele Scientific Institute, Milan, Italy; Division of Bilio-pancreatic endoscopy and Endosonography Division, San Raffaele Scientific Institute, Milan, Italy; Department of Gastroenterology and Alimentary Tract Surgery, Tampere University Hospital, Tampere, Finland; Department of Hepato-Gastroenterology, Cliniques Universitaires Saint-Luc, Brussels, Belgium; Department of Gastroenterology, University of Verona, Verona, Italy; Department of Gastroenterology. Complejo Hospitalario de Navarra, Pamplona, Spain; Department of Gastroenterology. Hospital Universitario de Santiago de Compostela, Santiago de Compostela, Spain; and Gastroenterology Department, Sant’Andrea Hospital, Roma, Italy. The reporting of this study was carried out in compliance with the STROBE guidelines for observational studies. Ethical approval was waived owing to the retrospective nature of the study.

### Inclusion and exclusion criteria

All patients with a presumed diagnosis of BD-IPMN and lacking any WF and/or HRS at the time of diagnosis, observed between January 2006 and December 2015, were included in the study.

A highly probable diagnosis of BD-IPMN was based on the presence of one or more dilated branch duct(s) communicating with a non-dilated MPD (3 mm or less) on high-resolution imaging, including MRI and/or CT with intravenous contrast and/or endoscopic ultrasonography (EUS)^4,5,9,12^.

A certain diagnosis of IPMN was based on cytological diagnosis obtained by EUS fine-needle aspiration (FNA) or fine-needle biopsy (FNB).

Patients were considered eligible for the study only if they had low-risk BD-IPMN, were under active surveillance, and had a minimum follow-up of 24 months. A family history of pancreatic adenocarcinoma (PDAC), considered as at least one first-degree relative affected by pancreatic cancer, was evaluated in the entire cohort. Patients aged less than 18 years, those with a history of major pancreatic surgery, and patients with WF and/or HRS at diagnosis were excluded from the analysis.

WF and/or HRS were based on 2012 guidelines^4^, and classified as follows.

WF were defined as cyst size over 30 mm, non-enhancing mural nodules, MPD 5–9 mm, acute pancreatitis, thickened enhanced cyst walls, and abrupt change in the MPD calibre with distal pancreatic atrophy. HRS were defined as major symptoms including jaundice, enhancing nodules, presence of malignant cells at cytology, and MPD size at least 10 mm. Pathological assessment included FNA, FNB, and the histopathological report.

### Study endpoints and data collection

The primary endpoint was development of WF and/or HRS during surveillance. Secondary endpoints included development of pathologically confirmed malignant IPMN, including both high-grade dysplasia (HGD) and invasive carcinoma, and occurrence of PDAC not associated with IPMN.

Demographic, radiological, pathological, and follow-up data were obtained from prospectively developed institutional databases. BD-IPMN was considered incidentally discovered in the absence of acute pancreatitis, jaundice or other symptoms including worsening or new-onset diabetes, steatorrhoea, unintentional weight loss, and non-specific abdominal pain. The latter symptom was described as pain without irradiation to the back and/or without increased serum amylase level. The site of IPMN was defined according to the anatomical location (head *versus* body–tail) and number of lesion(s) as diffuse when multiple cysts were present, and focal when a single cyst was diagnosed.

Surveillance was carried out using a combination of CT, MRI/magnetic resonance cholangiopancreatography, and EUS, but also included transabdominal ultrasonography, as stated in the Italian guidelines^12^. Of note, transabdominal ultrasonography was an imaging modality complementary to MRI or EUS. The date of diagnosis, date of each follow-up, and type of imaging were recorded. In patients with multiple lesions, the features of the largest cyst were included.

Indications for surgery were considered relative to when WF occurred or absolute when HRS developed during surveillance. Histology was assessed according to the 2010 WHO criteria^13^. Malignant IPMN included HGD, IPMN with invasive carcinoma, and PDAC not associated with IPMN.

The duration of surveillance was considered as the interval from diagnosis to the date of last follow-up, surgery or death. Death was categorized as pancreatic malignancy-related or pancreatic malignancy-unrelated.

Cyst size was categorized as below 15, 15–19, or at least 20 mm (but less than 30 mm); and MPD size between 0 and 5 mm was divided into two categories: 3 mm or less, or over 3 mm (but less than 5 mm).

### Statistical analysis

Curves showing the cumulative incidence of WF or HRS after the first examination (baseline examination at diagnosis) were drawn using the complement of the Kaplan–Meier method, and the log rank test was used to assess differences in incidence between subgroups of patients. Cox proportional hazards regression was used to identify factors associated with the development of WF or HRS. Variables considered as potential risk factors for the development or detection of WF or HRS included: centre expertise, sex, age, BMI, family history, smoking, alcohol consumption, diabetes, cyst site and size, MPD size, and symptoms. Factors with *P* ®< 0.050 in univariable analysis were entered into a multivariable model. Patients with MPD size 3 mm or less, cyst size less than 15 mm, and without symptoms at diagnosis were considered at very low risk, and subgroup analysis of these patients was undertaken. *P* < 0.050 was considered statistically significant. Statistical analysis was performed using SAS^®^ version 9.4 (SAS Institute, Cary, NC, USA).

## Results

### Patient characteristics

Between January 2006 and December 2015, 1153 patients were considered eligible for the study and 837 patients met all inclusion criteria (*Fig. S1*). Baseline characteristics are shown in *Table 1*. Some 10.0 per cent of patients reported symptoms at diagnosis, most often non-specific abdominal pain/discomfort. The median interval between follow-up imaging was 13.5 months. Median follow-up of the entire cohort was 4.8 years and 317 patients (37.9 per cent) had follow-up of more than 5 years.

**Table 1:** Baseline characteristics

	No. of patients* (*n* = 837)
**Sex**	
M	311 (37.2)
F	526 (62.8)
**Age (years)**	
Median (i.q.r.)	66 (58–72)
≤ 70	548 (65.5)
> 70	289 (34.5)
**BMI (kg/m^2^)**	
< 25	405 (48.4)
≥ 25, ≤ 30	329 (39.3)
> 30	103 (12.3)
**Family history**	
No	798 (95.4)
Yes	39 (4.6)
**Smoker**	
No	600 (71.7)
Yes	237 (28.3)
**Alcohol consumption**	
No	578 (69.1)
Yes	259 (30.9)
**Diabetes**	
No	729 (87.1)
Yes	108 (12.9)
**Disease focality**	
Unifocal	479 (57.3)
Multifocal	358 (42.7)
**Cyst site**	
Localized	689 (82.4)
Diffuse	148 (17.6)
**Cyst size (mm)**	
<15	496 (59.2)
15–19	151 (18.0)
≥ 20	190 (22.8)
**MPD size (mm)**	
≤ 3	280 (33.5)
> 3	512 (61.2)
Not specified†	45 (5.3)
**Symptoms**	
No	754 (90.0)
Yes	83 (10.0)
Non-specific abdominal pain	67 (8.0)
Weight loss	13 (1.5)
Steatorrhoea	5 (0.5)

* With percentages in parentheses unless indicated otherwise; †Not specified, but less than 5 mm. MPD, main pancreatic duct.

### Surveillance modality

**Fig. 1:**
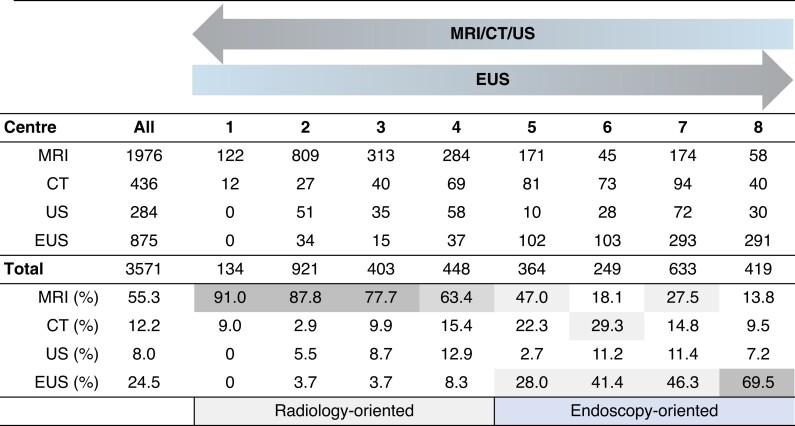
Number of examinations during follow-up until identification of worrisome features or high-risk stigmata US, ultrasonography; EUS, endoscopic ultrasonography.

The modality of surveillance differed between centres (*Fig 1*). A total of 3571 examinations were performed until the detection of WF or HRS. MRI (1976, 55.3 per cent) was the most common technique followed by EUS (875, 24.5 per cent), CT ( 436, 12.2 per cent,) and transabdominal ultrasonography (284, 8.0 per cent). Centres 1–4 (comprising 465 patients) were radiology-oriented, preferring MRI (80.2 per cent of all procedures) to EUS (4.5 per cent), whereas centres 5–8 (comprising 372 patients) were endoscopy-oriented, preferring EUS (46.5 per cent) to MRI (26.4 per cent).

### Development of WF/HRS and surgery during surveillance

**Fig. 2:**
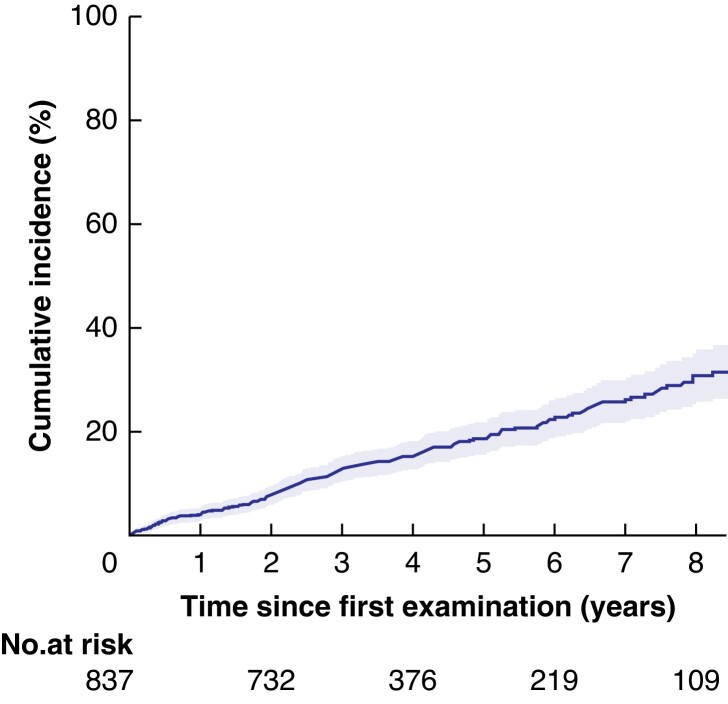
Development of worrisome features or high-risk stigmata during follow-up of 837 patients with branch-duct intraductal papillary mucinous neoplasms The shaded area represents the 95 per cent confidence interval.

Overall, 168 patients (20.1 per cent) developed WF (155) or HRS (13) during surveillance (*Fig. 2* and *Table 2*). Cyst size increasing to 30 mm or more was most common (48.2 per cent of patients with WG/HRS) followed by non-enhanced mural nodules (19.6 per cent), MPD over 5 mm and less than 10 mm (15.5 per cent), abrupt change in pancreatic duct (14.9 per cent), and wall thickening (10.1 per cent). Other findings were present in less than 10 per cent (pancreatitis in 6.5 per cent. enhanced solid component in 4.2 per cent, MPD 10 mm or larger in 3.0 per cent, and jaundice in 0.6 per cent of patients with WF/HRS). The cumulative incidence of WF/HRS was 18.7 (95 per cent c.i. 15.7 to 22.0) per cent at 5 years.

**Table 2:** Patients who developed worrisome features or high-risk stigmata

	No. of patients (*n* = 168)
**Worrisome features**	155 (92.3)
Pancreatitis	11 (6.5)
IPMN ≥ 30 mm	81 (48.2)
Abrupt change in pancreatic duct	25 (14.9)
Wall thickened	17 (10.1)
Non-enhanced mural nodes	33 (19.6)
MPD > 5 and < 10 mm	26 (15.5)
**High-risk stigmata**	13 (7.7)
Enhanced solid component	7 (4.2)
MPD ≥ 10 mm	5 (3.0)
Jaundice	1 (0.6)

Values in parentheses are percentages. IPMN, intraductal papillary mucinous neoplasm; MPD, main pancreatic duct.

Of 168 patients with WF/HRS, 132 (78.6 per cent) did not undergo surgery, and at the end of the observation they remained under surveillance without progression to pancreatic cancer. Of these patients, 6 per cent had HRS but EUS + FNA without positive cytology. They had associated co-morbidities and refused surgery because of increased risk of surgical complication.

Forty patients underwent surgery, including 36 who developed WF/HRS. HGD or invasive cancer was found in nine and nine patients respectively. The remaining four patients underwent pancreatectomy because of a family history of pancreatic cancer in the absence of a known genetic syndrome (3) or because of the patient’s decision (1). One patient in the former group had HGD. Histological findings are summarized in *Table S1*.

One patient with invasive carcinoma was found to have unresectable disease at laparotomy. This patient had been followed for BD-IPMN and a MPD duct size of 4 mm. After 1 year, this progressed to mixed-type IPMN with a pancreatic duct size of 12 mm and a solid pancreatic mass.

The rate of malignancy in the entire cohort was 18 of 837 (2.2 per cent) including invasive cancer in 9 (1.1 per cent). In the cohort of 168 patients who developed WF/HRS, the rate of malignancy was 10.1 per cent (17 patients); an invasive cancer was found in 5.4 per cent (9 patients).

### Risk factors for development of WF/HRS

**Fig. 3:**
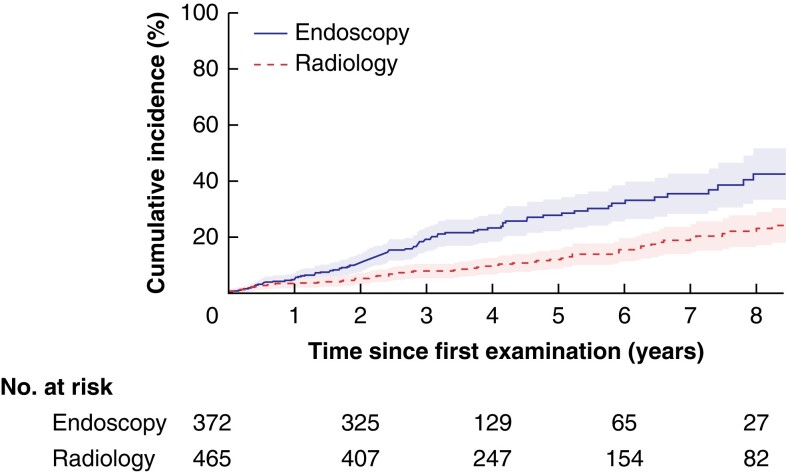
Detection of worrisome features or high-risk stigmata in endoscopy-oriented compared with radiology-oriented centres *P* < 0.001 (log rank test). Hazard ratio 2.46 (95 per cent c.i. 1.74 to 3.47) for endoscopy *versus* radiology, adjusted for site (diffuse, localized), cyst size (less than 15, 15–19, at least 20 mm), main pancreatic duct size (3 mm or less, more than 3 mm), and presence of symptoms. The shaded areas represent 95 per cent confidence intervals.

*Table 3* shows the results of univariable and multivariable analyses to identify predictors of development of WF/HRS. Independent predictors included localized IPMN site, cyst size, MPD size over 3 mm, and symptoms (*Fig. S2*). After adjustment for these factors, the detection of WF/HRS was increased in endoscopy-oriented centres compared with radiology-oriented centres (*Fig. 3*).

**Table 3:** Factors associated with the development of worrisome features or high-risk stigmata in univariable and multivariable Cox proportional hazards regression analysis

	Univariable analysis		Multivariable analysis	
	Hazard ratio	*P*	Hazard ratio	*P*
**Sex (F *versus* M)**	0.68 (0.50, 0.92)	0.01	0.74 (0.55, 1.01)	0.06
**Age (years)**				
50–59 *versus* < 50	1.23 (0.66, 2.31)	0.52		
60–69 *versus* < 50	1.30 (0.72, 2.32)	0.38		
≥ 70 *versus* < 50	1.56 (0.87, 2.79)	0.14		
**BMI**				
Underweight *versus* normal weight	0.91 (0.36, 2.29)	0.84		
Overweight *versus* normal weight	1.01 (0.65, 1.59)	0.95		
Obese *versus* normal weight	1.38 (0.69, 2.73)	0.36		
**Family history (yes *versus* no)**	0.79 (0.35, 1.78)	0.57		
**Smoker (yes *versus* no)**	1.58 (1.09, 2.27)	0.01		
**Alcohol consumption (yes *versus* no)**	1.34 (0.93, 1.93)	0.11		
**Diabetes (yes *versus* no)**	1.33 (0.87, 2.03)	0.19		
**Disease focality (multifocal *versus* unifocal)**	0.74 (0.54, 1.02)	0.06		
**Cyst site (diffuse *versus* localized)**	0.49 (0.31, 0.77)	0.002	0.43 (0.26, 0.68)	<0.001
**Cyst size (mm)**				
15–19 *versus* <15	1.93 (1.27, 2.95)	0.002	1.88 (1.23, 2.87)	0.004
≥20 (< 30) *versus* <15	3.47 (2.46, 4.89)	<0.001	3.25 (2.30, 4.60)	0.002
**MPD (> 3 (< 5) *versus* ≤ 3 mm)**	1.84 (1.21, 2.80)	0.004	2.17 (1.41, 3.34)	<0.001
Symptoms (yes *versus* no)*	2.28 (1.52, 3.42)	<0.001	2.29 (1.52, 3.45)	<0.001
**Abdominal pain (yes *versus* no)**	2.57 (1.65, 3.99)	<0.001		
**Weight loss (yes *versus* no)**	2.37 (0.87, 6.43)	0.09		
**Steatorrhoea (yes *versus* no)**	1.96 (0.48, 7.93)	0.35		

Values in parentheses are 95 per cent confidence intervals. *Includes abdominal pain, weight loss, and steatorrhoea.

**Fig. 4:**
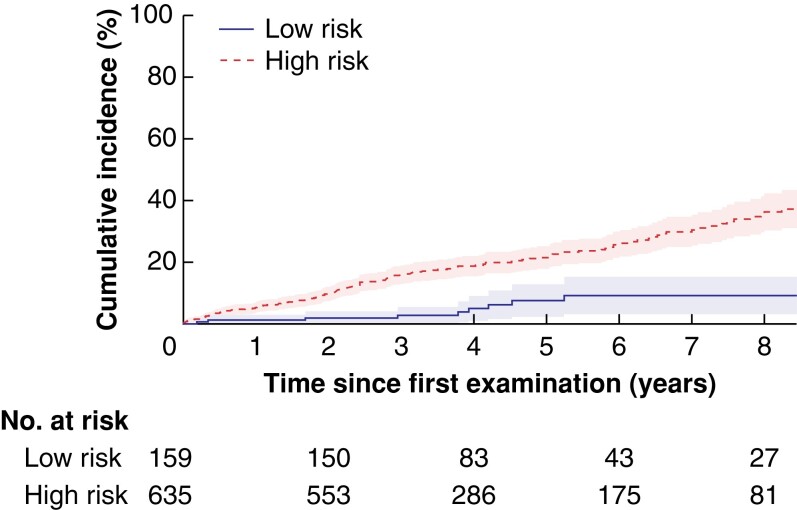
Cumulative incidence of worrisome features or high-risk stigmata during surveillance in low-and high-risk groups The low-risk group includes patients with a main pancreatic duct no larger than 3 mm, cyst size less than 15 mm, and no symptoms. The shaded area represents the 95 per cent confidence interval.

In the subgroup of 159 patients with MPD size 3 mm or less, cyst size less than 15 mm, and without any symptoms at baseline (median age 66 (range 19–83) years), 12 (7.5 per cent) developed WF/HRS, during 785 person-years of observation, corresponding to a rate of 1.5 per cent per year. This group of patients was defined as low risk based on clinicomorphological parameters. At 5 years, the cumulative incidence of WF/HRS was 7.7 per cent in this low-risk group compared with 21.5 per cent in remaining high-risk group (patients with MPD over 3 mm or cyst size at least 15 mm or with symptoms at diagnosis) (*Fig. 4* and *Table S2*).

## Discussion

Non-operative management of presumed BD-IPMN without WF or HRS is a safe strategy. The overall rate of malignancy during follow-up was low, with invasive cancer in 1.1 per cent of patients. Factors associated with the development of WF/HRS included localized IPMN, MPD size between 3 and 5 mm, and cyst size at least 20 mm. Centre expertise (or strategy) influenced the detection rate.

Several studies^10,11,14,15^ have identified cyst size over 15 mm as an independent predictor of development of WF/HRS in patients with low-risk BD-IPMNs undergoing surveillance. MPD dilatation is crucial for IPMN risk of malignancy. Most studies focused on the risk of malignancy when the MPD was between 5 and 9 mm or more than 10 mm in size^7,16,17^. Few studies addressed the size of a normal MPD (less than 5 mm) as a possible predictor of subsequent development of WF/HRS. An MPD growth rate of at least 0.2 mm/year is considered an independent predictor of WF/HRS^10^. The present study adds to the literature that MPD diameter of between 3 and 5 mm represents a risk factor for WF/HRS.

Symptoms were also a predictor of WF/HRS. Current guidelines^5,6^ consider acute pancreatitis, worsening or new onset of diabetes, and jaundice as WF/HRS. The present study showed that other symptoms should also be considered in patients with low-risk BD-IPMN, including steatorrhoea and non-specific abdominal pain. Salvia and colleagues^18^ noted a five-fold increase in the likelihood of steatorrhoea in patients with malignant main-duct IPMNs. Non-specific abdominal pain, however, is difficult to interpret and there might be recall bias among patients with more severe symptoms.

The follow-up strategy of the pancreatic centre influenced the detection rate. Radiology-oriented and endoscopy-oriented surveillance strategies were identified. The radiology-oriented centres followed patients longitudinally almost exclusively with radiology (90 per cent or more), typically MRI, whereas the endoscopy-oriented centres used EUS in up to half of patients. Endoscopy-oriented surveillance was mainly carried out by centres with a high level of expertise in advanced endoscopy. It is possible that EUS was considered as a second step during surveillance in selected patients with some changes in IPMN features (slight dilatation of the MPD or an increase in cyst size). The superiority of EUS in the detection of high-risk features in BD-IPMN is still debated. Although guidelines^5,6^ state that MRI is the best method by which to describe the communication between cysts and the ductal system, EUS seems more accurate in the identification of mural nodules as it provides the possibility of achieving a pathological diagnosis^19,20^. EUS may be considered when low-risk BD-IPMNs show clinical and radiological changes, even if WF/HRS are not yet present.

Patients with MPD size no larger than 3 mm, cyst size less than15 mm, and without any symptoms at baseline had a low risk of developing WF/HRS. This is concordant with the findings of Pergolini and co-workers^11^ and Crippa *et al*.^7^, who observed that cyst size below 15 mm was associated with a minimal risk of development of WF/HRS. These features may help tailor surveillance or even the decision to refrain from it, particularly for older patients or those with co-morbidity.

Limitations of the study included the retrospective design and heterogeneous follow-up strategies. Retrospectively, it was difficult to analyse the characteristics of abdominal pain. The better accuracy of EUS in detecting WF/HRS might have been a possible confounder in this study. The actual benefits of EUS over MRI should be explored in a prospective study.

## Supplementary Material

znac103_Supplementary_Data
