# Supplementary material for: Identification of patients with branch-duct intraductal papillary mucinous neoplasm and very low risk of cancer: multicentre study
Source: Br J Surg. 2022 May 3;109(7):617–22. doi: 10.1093/bjs/znac103 (PMC10364743; doi:10.1093/bjs/znac103)
Supplement: znac103_Supplementary_Data [file znac103_supplementary_data.zip › Supplementary_Table_1.docx]

**Table S1.** Baseline characteristics

|  | N (%) |
| --- | --- |
| **All eligible patients** | 837 (100) |
| **Sex** |  |
| Male | 311 (37.2) |
| Female | 526 (62.8) |
| **Age** |  |
| Median [IQR] | 66 [58-72] |
| ≤70 | 548 (65.5) |
| >70 | 289 (34.5) |
| **BMI** |  |
| <25 | 405 (48.4) |
| ≥25; <30 | 329 (39.3) |
| >30 | 103 (12.3) |
| Family history |  |
| Absent | 798 (95.4) |
| Present | 39 ( 4.6) |
| **Smoking** |  |
| No | 600 (71.7) |
| Yes | 237 (28.3) |
| **Alcohol** |  |
| No | 578 (69.1) |
| Yes | 259 (30.9) |
| **Diabetes** |  |
| No | 729 (87.1) |
| Yes | 108 (12.9) |
| **Focality** |  |
| Unifocal | 479 (57.3) |
| Multifocal | 358 (42.7) |
| **Site** |  |
| Localized | 689 (82.4) |
| Diffuse | 148 (17.6) |
| **Size** |  |
| <15mm | 496 (59.2) |
| 15-19mm | 151 (18.0) |
| ≥20mm (<30) | 190 (22.8) |
| **Wirsung size** |  |
| <3mm | 280 (33.5) |
| >3mm (<5) | 512 (61.2) |
| Unspecified size* | 45 ( 5.3) |
| **Symptoms** |  |
| No | 754 (90.0) |
| Yes | 83 (10.0) |
| *Unspecific abdominal pain* | *67 ( 8.0)* |
| *Weight loss* | *13 ( 1.5)* |
| *Steatorrhea* | *5 ( 0.5)* |

BMI: body mass index; IQR: Interquartile range; * unspecified but <5 mm
