# Supplementary material for: Identification of patients with branch-duct intraductal papillary mucinous neoplasm and very low risk of cancer: multicentre study
Source: Br J Surg. 2022 May 3;109(7):617–22. doi: 10.1093/bjs/znac103 (PMC10364743; doi:10.1093/bjs/znac103)
Supplement: znac103_Supplementary_Data [file znac103_supplementary_data.zip › Supplementary_Table_2.docx]

**Table S2.** Histology of patients who underwent surgery (n= 40)

| **Group, final histology** | **N** |
| --- | --- |
| **All patients** | **40** |
| Benign/Low-grade | 22 |
| High-grade | 9 |
| Invasive* | 9 |
|  |  |
| **Operated with WF or HRS** | 36 |
| Benign/Low-grade | 19 |
| High-grade | 8 |
| Invasive* | 9 |
|  |  |
| **Operated without WF or HRS** | 4 |
| Benign/Low-grade | 3 |
| High-grade | 1 |
| Invasive | 0 |

* includes 1 patient with HRS who underwent an exploratory laparotomy with unresectable disease.
