# Supplementary material for: Identification of patients with branch-duct intraductal papillary mucinous neoplasm and very low risk of cancer: multicentre study
Source: Br J Surg. 2022 May 3;109(7):617–22. doi: 10.1093/bjs/znac103 (PMC10364743; doi:10.1093/bjs/znac103)
Supplement: znac103_Supplementary_Data [file znac103_supplementary_data.zip › Supplementary_Figure_1.docx]

**Figure S1**: Flow chart of inclusion criteria

1153 patients considered

212 patients excluded because non available data on types of imaging during FU

1 patient was excluded because of history of acute pancreatitis

103 patients were excluded because not available data on IPMN characteristics (i.e. cyst size, MPD size, nodules etc)

941 patients were considered after exclusion

837 patients were enrolled in the present study
